# Vulnerability Assessment of IPv6 Websites to SQL Injection and Other Application Level Attacks

**DOI:** 10.1155/2013/946768

**Published:** 2013-12-26

**Authors:** Ying-Chiang Cho, Jen-Yi Pan

**Affiliations:** Department of Electrical Engineering, National Chung Cheng University, Chia-Yi 62102, Taiwan

## Abstract

Given the proliferation of internet connected devices, IPv6 has been proposed to replace IPv4. Aside from providing a larger address space which can be assigned to internet enabled devices, it has been suggested that the IPv6 protocol offers increased security due to the fact that with the large number of addresses available, standard IP scanning attacks will no longer become feasible. However, given the interest in attacking organizations rather than individual devices, most initial points of entry onto an organization's network and their attendant devices are visible and reachable through web crawling techniques, and, therefore, attacks on the visible application layer may offer ways to compromise the overall network. In this evaluation, we provide a straightforward implementation of a web crawler in conjunction with a benign black box penetration testing system and analyze the ease at which SQL injection attacks can be carried out.

## 1. Introduction

IPv6 was developed by Engineering Task Force [1] to solve the issue of insufficient number of addresses provided by the IPv4 protocol [2]. With the proliferation of internet enabled devise, the limits of IPv4 have been reached. IPv6 is composed of 128 bits, generating a total of 3.4 × 1038 addresses, which is 7.9 × 1028 times the address space of IPv4. Because of this greatly increased address space, most normal “war-dialing” type attacks [3, 4] are not feasible, and thus IPv6 offers an increased level of security versus IPv4. Therefore, most information security research with regard to IPv6 focuses mainly on the discussion of IP layer [5, 6] seeking to show that the underlying protocol is resistant to attack. However, this research ignores the overall nature of the Internet; that is, devices are inherently interconnected, and that once a vulnerable device can be identified linking information exists that allows one to identify other devices on the network. Therefore, it is trivial to traverse the links to attack the device of interest.

More specifically, most attacks are against organization rather than individual devices. Most of these organizations are connected to the internet in such a way where they can be searched via publically available search engines or a hyperlink structure can be traversed to reach them. After the initial web server or database has been identified and breached, other devices belonging to the organization can then be identified and compromised in turn, with the increased address space of IPv6 not making a difference. Thus, the increased address space provided by IPv6 does not offer any practical barriers to finding targets to attack.

To demonstrate this vulnerability, we will utilize the keyword search of publically available search engines such as Google, Bing, and Yahoo in conjunction with a web crawler with a black box penetration testing kit [7–9] to show how this can be done in principle.

## 2. Algorithm Principle

Briefly, the overall component of this system is a web crawler [10, 11] that takes an initial website, traverses the links on the front page, and tries to identify vulnerable links that can be exploited. This system also has a secondary component which utilizes search engines such as Google, Bing, and Yahoo to search for specific URLs that might be vulnerable to injection.

### 2.1. Web Crawling Algorithm

An effective web crawler needs to implement four key elements, a selection policy, a revisitation policy, a politeness policy, and parallelization scheme [12–15]. Briefly, the selection policy defines which sites that one will visit and includes aspects such as whether a link has been previously visited. A revisitation policy defines how often a link should be refreshed in order to detect changes. Politeness reflects the scheme by which a server is not overloaded with requests, and finally the parallelization scheme defines how the process can be parallelized for efficient searching.

**Figure 1:**
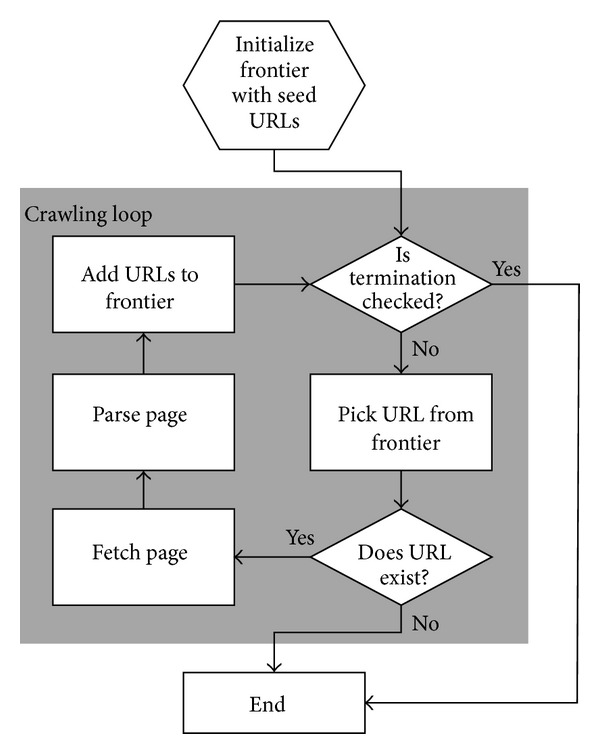
Web crawling flow chart.

The typical web crawler works via breadth first search [16], in which a frontier of unvisited links is first presented. These nodes are traversed, and a new frontier of unvisited sites is found after which the process repeats. One complication, however, is that to mark a site as visited, we normally rely upon a hashing protocol that functions on a canonical web address rather than just storing the address in its entirety. The overall process is given in Figure 1. However, one complication that needs to be addressed is that malicious websites or “spider traps” can be crafted so that web crawlers are trapped in an infinite loop [17]. Therefore, the hashing strategy must also take into account some of the content associated with the page.

### 2.2. Dynamic Analysis

Dynamic analysis identifies security problems by directly interacting with a functioning website. In other words, dynamic analysis relies on simulating user interactions with web pages, including interactions designed with potentially malicious intent. Because dynamic analysis uses a real website to find vulnerabilities in real time, found vulnerabilities are much more likely to be real than with static analysis, which has problems with detecting false positives [18, 19]. Black box testing determines whether a web application has a vulnerability by inputting testing data to the application and analyzing its response [9], as opposed to white box testing which focuses on source code parsing and analysis. White box testing tends to have lower efficiency because it does not factor in the dynamic interplay between the web server, application server, and database server [20]. Therefore, it is more common to use black box testing to more holistically analyze web application's vulnerability [21]. Penetration testing is a method to estimate the security of computer system or internet security by actively simulating attacks [22, 23]. This method analyzes all possible security weakness in the system, so the testing result is very valuable and convincing. The end product is not simply potential vulnerabilities but verified vulnerabilities and exploits. Honest testing result can form a bridge between developer and information security communities [24, 25].

### 2.3. Testing for SQL Injection

SQL injection [26–28] takes advantage of the process of web applications accessing databases with queries based on improperly validated user input. The website security mining system finds SQL injection attacks which can bypass firewall and identity authorization to control the database [29]. SQL injection can penetrate any type of database that relies on SQL, regardless of whether the underlying web application is written in ASP, PHP, or JSP as long as the program has a severe yet common logic error. Although there are well-known techniques to combat SQL injection attacks [30, 31], they are still quite common, and, therefore, there has been much interest in developing methods to inspect web applications and detect these vulnerabilities [29, 32, 33].

### 2.4. Testing for Brute Force

A complete dictionary file is important to our research. The web crawler we designed will detect the pages with weak password [34], and hence the detection ability [35, 36] will be decided on quality of the dictionary file. In particular in case of crawling database form, ineffective dictionary file causes slow crawling while insufficient dictionary file fails to determine whether the webpage's password detected reaches the information security standards [37, 38]. With thousands of real experiments, our system refers to many related literatures [9, 39–43] and web vulnerability scanners practically applied. We provide function of adjustable parameters to handle different environment through flexible adjustment (for instance, Http versus FTP, whether complying with protocol of robots.txt, priority of attacks facing numerous database, the depth of crawling a webpage and whether detecting broken links, etc.). However, we do not focus on these issues here.

### 2.5. The Security Issues from IPv4 to IPv6

**Figure 2:**
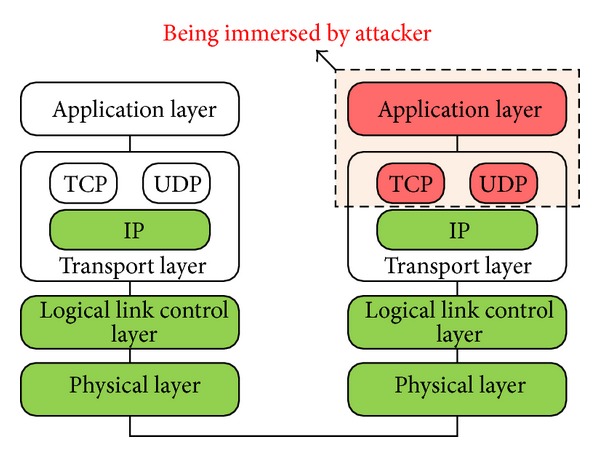
Application attack.

There are three techniques to transform IPv4 to IPv6 addresses which are dual stack, tunneling, and translation [44]. Most of the security research with respect to IPv6 has focused upon these translation layers as well as in the authentication and encryption of the individual data packets [45–48]. The primary concern that security researchers have tried to address is the problem of incorrect redirection and spoofing. However, it should be noted that the majority of attacks against the current IPv4 infrastructure do not occur at the transport layer but rather at the application layer [49] and that these attacks still apply to IPv6. For instance, attacks such as sniffing, application layer attacks [50], rogue devices, man-in-the-middle attacks, and flooding are still applicable. Both IPv4 and IPv6 are vulnerable when facing application layer attacks [51], as shown in Figure 2 [52].

Among many application level attacks methods, SQL injection is the most well known. Furthermore, because it attacks databases which may store information related to accounts and authentication, they are an attractive target to hit. In our evaluation we combine the discovery module which utilizes web crawling with black box penetration methods [53] to implement a system which is called website security mining system. It has two modules and six functions. The modules are the dynamic scanning module and static mining module. The functions are the syntax analysis function, search engine assistant function, hidden page searching function, vulnerability updating function, specific file searching function, and multidomain lookup with single IP function. The experimental targets are each country's IPv6 official website. We use the system to crawl each website 24 hours and gather statistics to each site's found e-mails and injectable URLs to compare the security protection done in each country's IPv4 website.

## 3. System Implementation 

In order to inspect if information stored on the web presents a security risk, this research combines a web crawler, like those used in search engines, with the concept of application vulnerability inspection, specifically black box and penetration testing. The end product is the website security mining system, a tool to evaluate a website's security. This system can be separated into two main modules which are the Static Mining module and the Dynamic Scanning module. The Static Mining module inspects a specific website's robots.txt, E-mails, potential SQL injection URLs, files, and broken links. The Dynamic Scanning module uses the system's vulnerability-inspecting function by typing keywords into a search engine's query box to inspect many websites.

Both the Static Mining and Dynamic Scanning modules leverage the system's vulnerability inspecting function, which has two parts: known website vulnerability inspection and SQL injection inspection. The former compiles a database of open source website vulnerabilities into an XML file which is used to inspect the website to see whether it has the same vulnerability. Algorithm 1 is the format of an XML file. The bug file parameter is a base64 hash and other parameters are converted from the open source website vulnerabilities database. Our system updates its vulnerability database by adopting new vulnerabilities that have been announced on the Exploit Database regularly [54]. By updating the vulnerability database, we can ensure that the vulnerability samples are always updated, similar to how antivirus software regularly updates its virus database.

The website security mining system can find vulnerabilities in a variety of database engines, specifically MS-SQL, MySQL, Access, Oracle, Postgresql, and Sqlite. The steps to identify SQL injection vulnerabilities are as follows. First, an injectable point must be identified by inspecting the website for places where user input may be used in SQL queries. If such an injectable point is found, then further tests are conducted to identify the specific type of database engine. To do this, we take advantage of how different databases use different function names for certain tasks. For example, MS-SQL and MySQL use len( ) to calculate length; however, Oracle uses length( ) to do it. In other words, when you use len(“s”) = 1 to test and receive a normal response, that means the target website uses MS-SQL or MySQL. On the other hand, if this does not work, then the database might be Oracle or other types of database. There are several other functions that can help us determine what the database is. After getting the database's type, we find table and column names and finally get the database's data.

The website security mining system can run on any operating systems that are supported by Java. We describe the two basic modules in more detail below.

### 3.1. Static Mining Module

The Static Mining module runs depth mining on a specific website. There is an option to determine whether you want to follow the website's robot.txt rules. Robot.txt [55] is an ASCII-encoded file stored in the website's root directory that lists files that can and cannot be accessed by search engine crawlers. There is no official standards body or RFC for the robots.txt format. It was created by consensus and ultimately serves only as a recommendation for crawlers, so it cannot protect the website's private data completely [56]. Other functions of the Static Mining module are identifying e-mail information, potentially injectable URLs, downloadable files, and broken links, which may contain private information.

The Static Mining module starts with a specific web site and then collects all the related pages from it using a breath-first search algorithm. The system assumes that web pages have close relations to the original web page if the link distance is within a certain range [57–59], so it will fetch all links inside the original page then iterate through all of those URLs to fetch all links within them. This type of method can be easily parallelized to improve fetching speed. After files are downloaded by the web crawler, an HTML parser process extracts pages' URLs and then adds it into the URL queue. Also, the system will call vulnerability inspecting process to inspect URLs, checking whether it has potential vulnerabilities or not.

**Figure 3:**
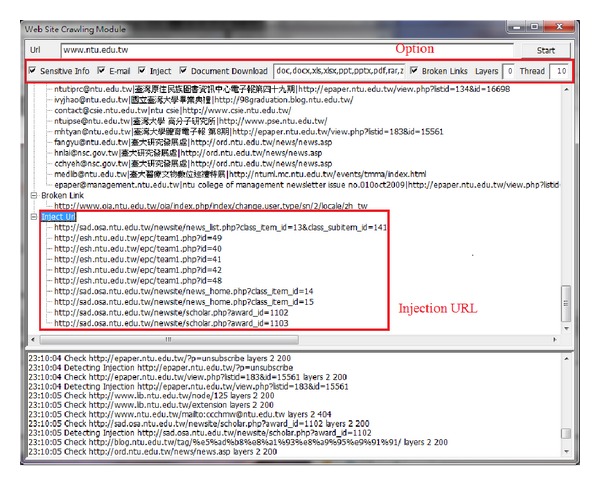
Static Mining module.

**Figure 4:**
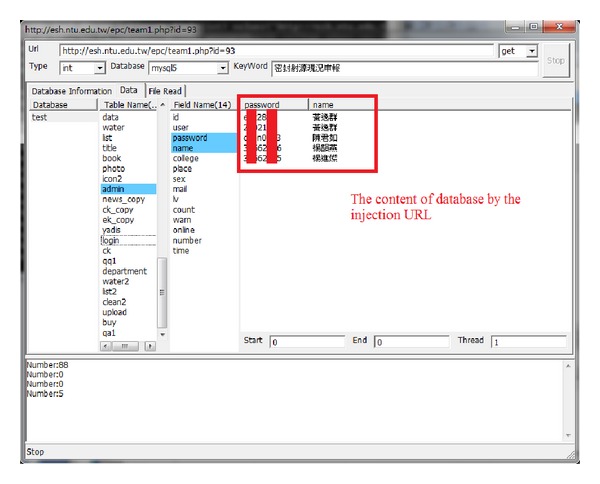
Database content found by injectable URLs.

Figure 3 shows the process of mining a college's website [60] by our system. Several injectable URLs were found and by exploiting these vulnerabilities we were able to retrieve the database information shown in Figure 4.

**Figure 5:**
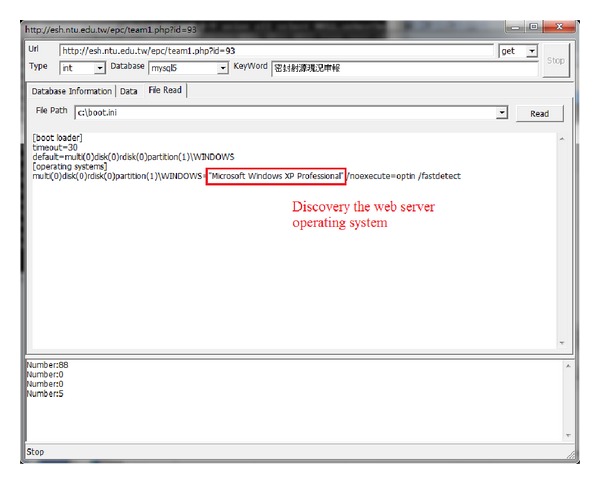
Host operating system.

Additionally, we determined that the operating system (OS) of the host was “Microsoft Windows XP Professional,” as shown in Figure 5, which could open up the possibility for further OS-based exploits.

### 3.2. Dynamic Scanning Module

The most popular search engines today are Google, Yahoo, Baidu, Microsoft Bing, NHN, eBay, Ask.com, Yandex, and Alibaba. With the help of search engines, we can find billions of web pages and their URLs. Our system inspects these websites to determine whether they have vulnerabilities by analyzing the results retrieved from search engines. Our system supports the kinds of query syntaxes used in modern search engines. After you input the keywords, the system can find all related web pages and inspect whether they are at risk for vulnerabilities or not.

**Figure 6:**
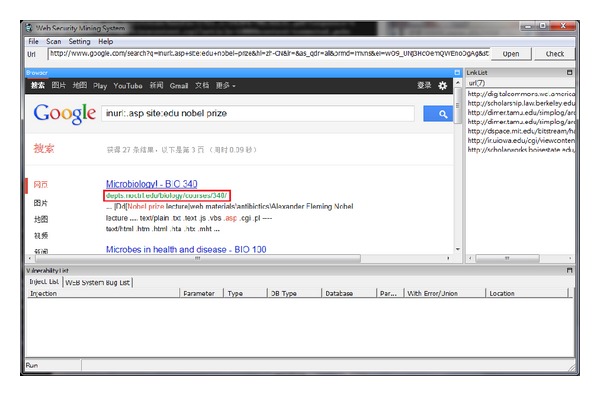
Work on Google search engine.

**Figure 7:**
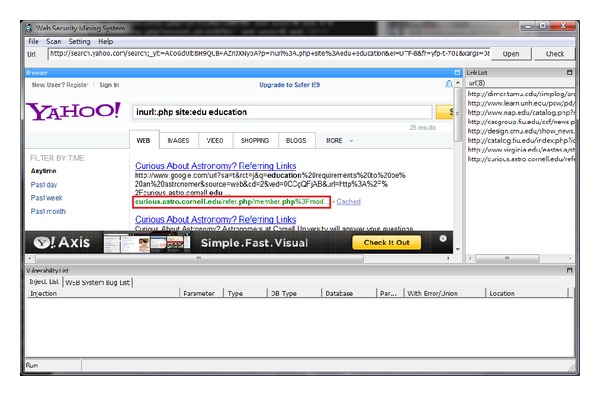
Work on Yahoo search engine.

**Figure 8:**
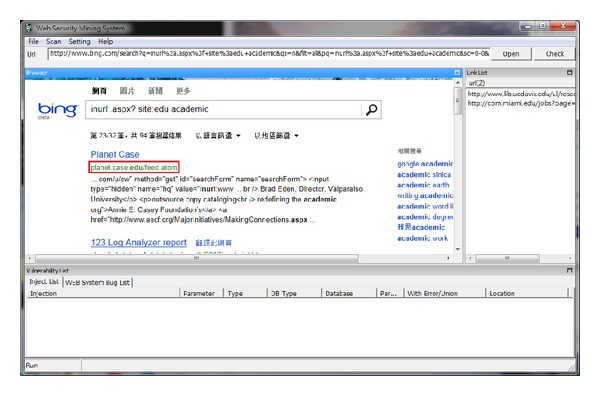
Work on Bing search engine.

Figures 6, 7, and 8 show the different query syntaxes used in different search engines. For Google, it is “inurl:.asp? site:edu nobel prize”; Yahoo is “inurl:.php? site:edu education”; Bing is “inurl:.aspx? site:edu academic”.

**Figure 9:**
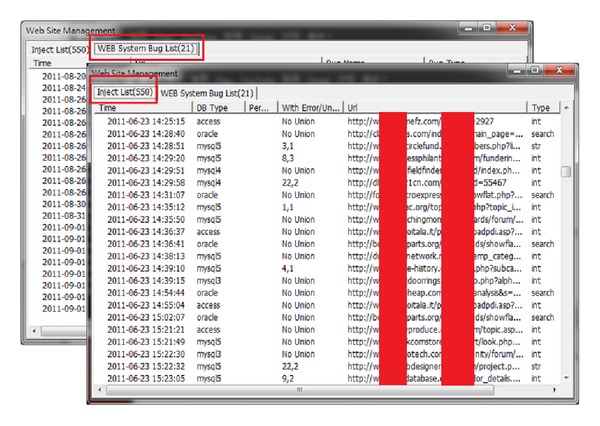
Dynamic scanning results.

This research used the same command, inurl:.asp? *|*.jsp?*|*.php?*|*.aspx? site:com new, to search the ten most popular search engines. 800 web pages were retrieved from each search engine. We found 550 SQL injectable URLs and 21 known website vulnerabilities out of this total of 8000 web pages, which are shown in Figure 9 below. This highlights the fact that SQL injection problems are still very severe on the internet.

Despite SQL queries injection, this system provides functions of backend systems detection [61], session hijacking [62], Cookie poisoning [63], form manipulation [64], URL parameter tampering [65], HTTP header modification [66], bypassing intermediate forms in a multiple-form set [61], cross-site scripting [67], third party software misconfiguration [61], forceful browsing [43], and several security tests related to application layer. However, we do not focus on these issues here.

## 4. Experiments

We designed two experiments to test the security situation of IPv6's website on the internet. Experiment 1 uses WSMS's Dynamic Scanning module to compare the numbers of injectable URL in each IPv4 and IPv6 website. Experiment 2 uses WSMS's Static Mining module to crawl the websites that have been authorized by IPv6 forum [68], which can help us realize the situation of e-mail leakage and database leakage in IPv6's websites.

### 4.1. Experiment 1

We constructed WSMS in the pure IPv4 environment; it will show “getaddr info failed URL Error” message and stop if it crawled IPv6's website. In the case where we wish to explore IPv6 addresses, the converse will be true; that is, getaddr info URL failed will be returned for IPv4 address. We uses Dynamic Scanning module to search these four type web pages (asp/aspx/php/jsp) in three different search engines (Google/Yahoo!/Bing) with “world peace” as the keyword. The statements which we type in Google, Yahoo!, and Bing are shown as

 Google => inurl:asp? world peace
 Yahoo! => world peace asp?
 Bing => world peace asp?

**Figure 10:**
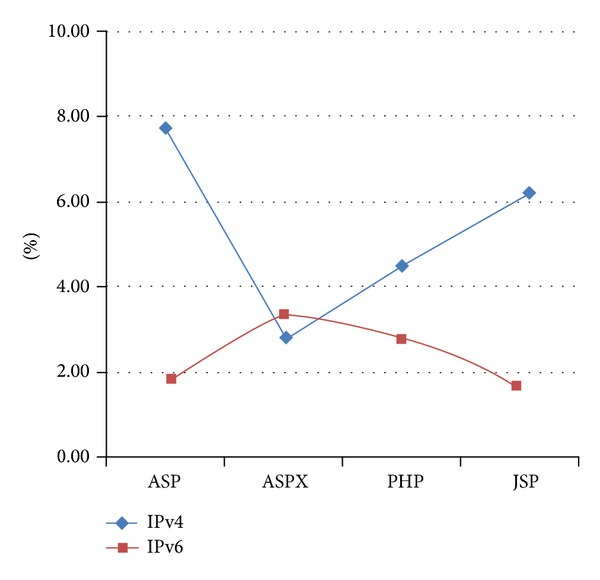
Ratio of injectable URL.

We assumed that *X* represents the pure IPv4 web pages that contain world peace, *Y* represents the number of URLs that have been inspected by WSMS, and *Z* represents the number of URLs that are injectable. The operating process and data results are shown by Tables 1, 2, and 3 (also see Figure 10).

As shown in Table 4 analyzed through type of website, results of Analysis of variance (ANOVA) [69] (*P* value = 0.873 > 0.05) showed that the type of website has no significant contribution to the rate of vulnerability in either IPv4 or IPv6. In Tables 5 and 6 analyzed through type of network environment, we knew that IPv4 and IPv6 have great difference in the prevalence of URL injection from results of independent samples *t*-test. In other words, upon the confident level of 95%, the average prevalence rate of injectable URL of IPv4 (5.55%) is larger than IPv6 (2.94%). However, the reasons for above statement remain unclear. We make hypothesis that the main reason that IPv6 sites have better security than IPv4 sites is because the IPv6 sites are newer and the programmers of these sites are more cognizant of vulnerabilities such as SQL injection and have already taken steps to mitigate these issues.

The experiment result shows that the number of inspectable URLs in Google is the highest above all because it supports the function of parameter “inurl.” In IPv4's situation, ASPX has the least injection problem while ASP gets the worst outcome. In IPv6's situation, JSP has the least injection problem while ASPX performs poorly. In general, ASPX has much fewer problems and ASP has most problems. From Tables 7 and 8, IPv4 and IPv6 have great difference in the virus number detected in view of types of website, while from the result of Chi-square test, we discovered that ASP website accounts for 34.6% of virus detected in IPv4, followed by JSP with 29.1%; these two kinds of website perform better in IPv6 environment. In IPv6, PHP accounts for 38.9% of virus detected, followed by ASPX with 27.8%, and these two kinds of website perform better in IPv4.

### 4.2. Experiment 2

We constructed WSMS on the pure IPv6 environment, using Static Mining module to crawl twenty websites which were randomly selected from twenty regions from the IPv6 forum, and then we gathered the amount of E-mail address and injectable URL again (shown in Table 9); we see a significant number of websites in both IPv6 and IPv4 that are susceptible to attack, with IPv6 showing a lower level of vulnerability.

From the above two experiments, we can see that the migration to IPv6 still leaves a great deal of vulnerabilities present in the application level infrastructure with a great deal of vulnerabilities still existing. These vulnerabilities while known can represent the initial springboard for more targeted attacks.

## 5. Conclusion

One of the messages from this evaluation is that with respect to the majority of the attacks that are commonly used, IPv6 does not offer any increased level of security versus IPv4. This is not surprising given the fact that the application layer attacks bypass the majority of the security infrastructure built into IPv6. Therefore, the results of this evaluation are hardly surprising. However, one interesting consequence of IPv6 is that given the large address space, it becomes more difficult to identify where malicious attacks are coming from due to the fact that an attacker no longer has to be tied to a small number of IP addresses but instead has a much larger pool with which to hide. Without the need to be readily discoverable by the general public, this level of anonymity becomes a much stronger weapon for the attacker than it is for the defender. That being said, with a better understanding vulnerability, we see that newer systems are much more robust than legacy systems. This perhaps is the most important result of this paper.

The website figures sampled from the experiment can prove that, even though the injection problem of IPv6 website is less than the IPv4 one, IPv6's security protection on the transport layer does nothing to mitigate shortcomings on the application layer. Therefore, the programming habit [26, 70, 71] of the programmer is still critical. We all know that the information stored online is not one-hundred-percent safe, but one of the measures that an end user can take is to increase the complexity of its password setting [72, 73]. As for the server database side, the plain text password should be encrypted [74] before it is stored in the database, so that the hacker will not obtain easily authentication tokens when he breaks in the website and obtains the content of the database. The empirical measures show that aside from the website programming logic and database security management, the encrypted storage of the data is also important.

## Figures and Tables

**Algorithm 1:**
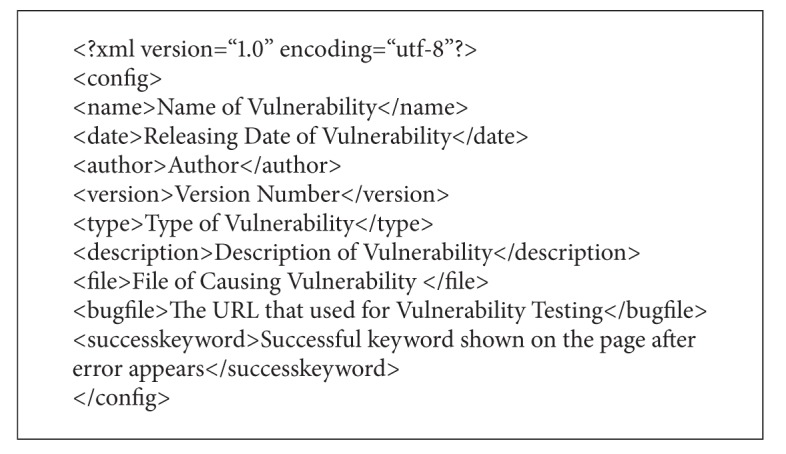
XML format.

**Table 1:** Pure IPv4 website detection statistics.

(*X*, *Y*, *Z*)	Google	Yahoo!	Bing
ASP	(831, 292, 22)	(558, 123, 7)	(575, 157, 15)
ASPX	(875, 286, 8)	(559, 68, 5)	(623, 153, 1)
PHP	(917, 311, 15)	(741, 177, 7)	(655, 220, 10)
JSP	(866, 290, 12)	(501, 171, 13)	(571, 152, 12)

**Table 2:** Pure IPv6 website detection statistics.

(*S*, *T*, *U*)	Google	Yahoo!	Bing
ASP	(482, 222, 4)	(504, 25, 1)	(517, 22, 0)
ASPX	(368, 97, 2)	(502, 31, 1)	(506, 18, 2)
PHP	(520, 175, 3)	(607, 45, 2)	(639, 29, 2)
JSP	(128, 36, 0)	(516, 16, 0)	(546, 6, 1)

**Table 3:** Ratio of injectable URL.

	ASP	ASPX	PHP	JSP
IPv4	7.69%	2.76%	4.52%	6.04%
IPv6	1.86%	3.42%	2.81%	1.72%

Average	4.78%	3.09%	3.67%	3.88%

**Table 4:** ANOVA.

	SS	DF	MS	*F*	*P*
Ratio of injectable URL					
Between	0.001	3	0.000	0.232	0.873
Inner	0.023	20	0.001		
Sum	0.024	23			

**Table 5:** Group statistics.

	Web page	Number	Mean
Ratio of injectable URL	IPv4	12	0.05545133
	IPv6	12	0.02937988

**Table 6:** Independent samples test.

	Levene's test for equality of variances		*t*-test for equality of means		
	*F* test	*P*	*t*	DF	*P* (2-tailed)
The ratio of injectable URL	0.368	0.550	2.147	22	0.043

**Table 7:** Page ∗ type cross-tabulation.

	Type				Sum
	ASP	ASPX	PHP	JSP	
IPv4 page					
Amount	44	14	32	37	127
Ratio	34.6%	11.0%	25.2%	29.1%	100.0%
IPv6 page					
Amount	5	5	7	1	18
Ratio	27.8%	27.8%	38.9%	5.6%	100.0%
Sum					
Amount	49	19	39	38	145
Ratio	33.8%	13.1%	26.9%	26.2%	100.0%

**Table 8:** Chi-square test.

	Value	DF	Asymp. Sig. (2-tailed)
Pearson Chi-square	10.329^*a*^	3	0.016

*a*: 3 cells (37.5%) have expected count less than 5. The minimum expected count is 2.24.

**Table 9:** Experiment 2 result output.

Region/country	Tags	URL	MAIL disclosure amount	Inject URL amount
Mexico	Enterprise site	http://arteria.com.mx	91	0
Brazil	IT site	http://bgp.net.br	169	0
Denmark	Other	http://mirrors.dotsrc.org	13	0
Russia	Other	http://rusnavi.org	46	0
Belgium	Government site	http://www.awt.be	565	3
Argentina	Education site	http://ipv6solutions.com.ar	0	0
Spain	Education site	http://www.cba.upc.edu	557	0
Britain	Education site	http://www.ecs.soton.ac.uk	1697	0
Canada	Personal site	http://www.ampedcanada.com	0	0
America	Not-for-profit cooperative site	http://www.fairfaxcirclechurch.org	12	0
Germany	Other	http://www.das-labor.org	71	0
New Zealand	Other	http://www.geekzone.co.nz	97	0
Italy	Government site	http://www.imaa.cnr.it	33	0
India	Government site	http://www.nixi.in	77	0
Japan	Personal site	http://www.robata.org	82	0
Taiwan	Education site	http://www.yfp.ks.edu.tw	3	0
Thailand	Education site	http://ns6.cpe.rmutt.ac.th	0	0
China	Education site	http://www.zzrvtc.edu.cn	430	59
Switzerland	Other	http://www6.itu.int	1691	13
Poland	Education site	http://zsp6siedlce.pl	3	0
